# It Can Wait

**Published:** 1887-09

**Authors:** 


### IT CAN WAIT.

The respected editor of the *Journal of the American Medical Association*, in the number for Aug. 6th, reviews the editorial in our last number upon "Medical Recognition," and indulges in some comments upon it. We have very positive ideas as to who is decidedly "mixed," and in the near future propose to devote a little space to the consideration of the question. But at present there is one duty which should absorb the energies of all of us, and that is the labor of making of the International Congress meeting a grand success. Until that is passed, controversies are not in good taste.

